# Edwin Leonard Lees

**Published:** 1949-04

**Authors:** 


### Obituary

EDWIN LEONARD LEES, M.D., C.M. Edin.

Dr. Leonard Lees, who died on January 10th, 1949, was born at St. Andrews in 1863, and received his medical training at Edinburgh, where he was a student under Dr. Joseph Bell, the prototype of Sherlock Holmes. He qualified in 1885 and then came to Bristol, where he became first partner and later successor to Dr. Coleman in Redland. He took his M.D. in 1890. He was appointed House Surgeon to the Children's Hospital, then Registrar and in 1896 Physician: he held this appointment until 1905 when he was appointed Consultant Physician. During the whole of his life, until he retired in 1946, he was in busy general practice, and endeared himself to all whom he met, patients and colleagues alike. He was an enthusiastic pioneer motorist and a keen golfer. In 1890 he married Miss Alma Ehlers: to her and to their family we tender our sincere sympathies.

